# Three-dimensional reconstruction of a small piece of Ce-doped lithium glass scintillator of an optical fiber-based neutron detector using microcomputed tomography technique

**DOI:** 10.1093/jrr/rraf048

**Published:** 2025-07-29

**Authors:** Akihisa Ishikawa, Mariko Segawa, Yosuke Toh, Kenichi Watanabe, Akihiko Masuda, Tetsuro Matsumoto, Atsushi Yamazaki, Sachiko Yoshihashi, Akira Uritani, Hideki Harano

**Affiliations:** Research Group for Nuclear Sensing, Nuclear Science and Engineering Center, Japan Atomic Energy Agency, 2-4 Shirakata, Tokai-mura, Ibaraki 319-1195, Japan; Research Group for Nuclear Sensing, Nuclear Science and Engineering Center, Japan Atomic Energy Agency, 2-4 Shirakata, Tokai-mura, Ibaraki 319-1195, Japan; Research Group for Nuclear Sensing, Nuclear Science and Engineering Center, Japan Atomic Energy Agency, 2-4 Shirakata, Tokai-mura, Ibaraki 319-1195, Japan; Department of Applied Quantum Physics and Nuclear Engineering, Kyushu University, 744, Motooka, Nishi-ku, Fukuoka 819-0395, Japan; National Metrology Institute of Japan (NMIJ), National Institute of Advanced Industrial Science and Technology (AIST), 1-1-1 Umezono, Tsukuba, Ibaraki 305-8568, Japan; National Metrology Institute of Japan (NMIJ), National Institute of Advanced Industrial Science and Technology (AIST), 1-1-1 Umezono, Tsukuba, Ibaraki 305-8568, Japan; Department of Applied Energy, Graduate School of Engineering, Nagoya University, Furo-cho, Chikusa-ku, Nagoya, Aichi 464-8603, Japan; Department of Applied Energy, Graduate School of Engineering, Nagoya University, Furo-cho, Chikusa-ku, Nagoya, Aichi 464-8603, Japan; Department of Applied Energy, Graduate School of Engineering, Nagoya University, Furo-cho, Chikusa-ku, Nagoya, Aichi 464-8603, Japan; National Metrology Institute of Japan (NMIJ), National Institute of Advanced Industrial Science and Technology (AIST), 1-1-1 Umezono, Tsukuba, Ibaraki 305-8568, Japan

**Keywords:** neutron, scintillator, neutron detector, microcomputed tomography (micro-CT)

## Abstract

An optical fiber-based neutron detector is a real-time neutron monitor for an intense neutron field. A small piece of neutron scintillator, such as Ce-doped lithium glass (Li-glass), used in the detector has a random shape with a grain size of 200–400 μm. This causes shape-dependent effects on the detector response. However, it is difficult to control or determine its shape due to its small size. Here we propose a technique to characterize the fine structure of a small piece of scintillator using a microcomputed tomography (CT) system. To verify accuracy, the mass calculated based on the density of Li-glass and the volume extracted from the obtained CT image was compared to the mass measured in advance using an electronic balance. In the obtained CT images, the fine shape of the small piece of Li-glass was clearly visible, and no false signals from the surrounding components were observed. The calculated mass was in good agreement with the measured value, however, when the total number of projection images was 2000, a slight underestimation was observed. This was mitigated by increasing the number of projection images, and the difference between the calculated and measured mass was 1.6% when the number of the projection images was 3141. This was equivalent to the uncertainty of the measured mass. The proposed technique will be useful when high accuracy is needed, such as for medical applications.

## INTRODUCTION

Neutrons are widely used in various fields such as material life science [1, 2], medical applications [3, 4], and homeland security [5, 6]. There are several requirements for a neutron detector, especially in a high-intensity neutron field, such as low sensitivity to coexisting γ-rays and a wide dynamic range of the neutron counting rate. We have developed an optical fiber-based neutron detector for use as a real-time neutron monitor for an intense neutron field [7, 8]. A small piece of neutron scintillator, such as Eu-doped LiCaAlF_6_ [9, 10] or Ce-doped lithium glass (Li-glass), with a grain size of ⁓200–400 μm is used in the detector. This enables the lowering of the sensitivity to coexisting γ-rays while maintaining sensitivity to neutrons [11], and mitigates the impact of fluctuation in the radiation field to be measured. However, the small piece of neutron scintillator has a random shape, which makes it difficult to quantify or correct the shape-dependent effects on the detector response. Furthermore, it is not practical to process it into a specified shape because of its small size. Although the shape-dependent effects on the detector response are relatively diminished due to its small grain size, the detector is expected to exhibit the self-shielding effect and the directional dependence of the response. To date, a quantitative evaluation of these effects has not been possible.

A Li-glass transparent composite scintillator was developed by mixing Li-glass powder and ultraviolet curable resin [12]. This technique enabled the shape and amount (i.e. neutron sensitivity) of the small scintillator used in the optical fiber-based neutron detector to be roughly controllable. In the measured pulse height spectrum for a ^252^Cf neutron source, however, the observed neutron peak of the Li-glass transparent composite scintillator had a lower pulse height, and a bigger width, compared to that observed using a bulk Li-glass scintillator. This was due to the decreased efficiency and uniformity of the photon collection stemming from the turbidity of the scintillator. These changes might be disadvantageous for the optical fiber-based neutron detector because they would worsen the detector properties, such as n-γ discrimination based on pulse height. Additionally, even if some degree of shape control of the scintillator is possible, the actual shape of the scintillator in the fabricated detector must be evaluated to correct the shape-dependent effects on the detector response. In addition, the detailed shape and geometry of the scintillator may affect the light collection efficiency of the detector.

According to the ICRU24 report, changes of 7%–10% in the administered dose affect the tumor control probability, and the dose delivered to the patient must be measured with an overall dose uncertainty of ˂5% in X-ray radiotherapy [13]. Similarly, doses in radiotherapy using neutrons, such as boron neutron capture therapy (BNCT), should also be measured with high accuracy. Provided that the detector count has a statistical uncertainty of 1%, the systematic uncertainty of the detector must be ˂4.9% to determine the neutron fluence with an uncertainty of ˂5%. Although the optical fiber-based neutron detector can be a promising candidate for this high accuracy neutron measurement, the micro shape of the small piece of scintillator is expected to affect the detector response. To determine the fine structure of the small piece of the scintillator is important for understanding the detector response, and is essential for correction of the detector response to diminish the systematic uncertainty. However, the technique to finely evaluate the scintillator shape has not yet been established. Thus, the purpose of this study is to propose a technique to finely determine the micro shape of the small piece of scintillator, and to verify its accuracy by comparing the mass derived using the technique and that measured by electronic balance.

## MATERIALS AND METHODS

### Optical fiber-based neutron detector

**Fig. 1:**
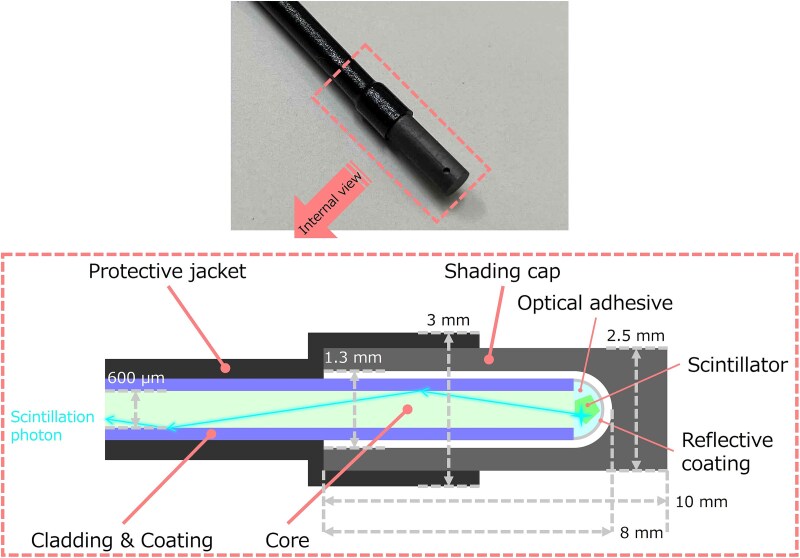
Schematic diagram of the optical fiber-based neutron detector.

The schematic diagram of the optical fiber-based neutron detector is shown in Fig. 1. A small piece of the neutron scintillator is attached to the tip of the optical fiber (>10 m) using optical adhesive. It is coated with a reflective coating and covered with a shading cap. The fiber is covered and shaded with a protective jacket except for the tip. The detector has a maximum external diameter of 3 mm.

### Sample preparation and mass measurement

In this study, samples of the optical fiber-based neutron detectors were fabricated using small pieces of Li-glass scintillator with a grain size of 212–400 μm. Li-glass, used in our detector [15], is known to have a relatively fast decay time constant of 75 ns [14]. This is advantageous in high counting rate situations. To investigate the differences due to the size and composition of Li-glass, three different samples were prepared. 95% ^6^Li-enriched Li-glass (GS20, Scintacor, Cambridge, England) was used in two of the samples (named ‘En-L’ and ‘En-S’), and natural isotopic ratio ^6^Li-glass (GS10, Scintacor, Cambridge, England) in another (named ‘Nat’). They had the maximum emission wavelength of 395 nm. The mass density of Li-glass is 2.50 g/cm^3^ [14]. It should be noted that the small piece scintillator used in this study was prepared by grinding a bulk of the scintillator, and was considered to have the mass density equivalent to that of the bulky scintillator. Multimode quartz optical fiber (FP600URT, Thorlabs, New Jersey, United States) with a length of 10 m was used. The FP600URT core had a diameter of 600 μm and an attenuation of nearly 70 dB/km, which led to a transmission loss of 15% at 10 m. Its numerical aperture was 0.5, which meant that the scintillation photons emitted within the critical angle of 30° were collected and led to the transmission mode of the core. Ultraviolet curable resin (NOA63, Norland, New Jersey, United States) was used as the optical adhesive. NOA63 had a transmittance of ˃99% at 395 nm. Correction fluid, the main component of which was titanium (IV) oxide (TiO_2_), was used as the reflective coating. The shading cap, which was made of graphite, had a length of 10 mm and an external diameter of 2.5 mm. It had a well structure with an inner diameter of 1.3 mm and a depth of 8 mm to insert the tip of the fiber. It also had a dimple with a diameter of 0.5 mm on its lateral side to identify the direction of the detector to the radiation source. Heat shrinkage tube (TC26, HellermannTyton Co., Ltd., Tokyo, Japan) was used as the protective jacket and the shading material. Although the structure of the detector for actual use was as shown in Fig. 1, it should be noted that the shading cap and protective jacket were not included in the samples in this study to ensure external visibility during the experiments.

To assess the volume of Li-glass used in the samples, the mass of the small pieces of Li-glass was measured in advance using an electronic balance (XP6, Mettler Toledo, Greifensee, Switzerland) with a mass readability of 1 μg according to the following steps. First, the value indicated with no sample was read as background (= 0 μg). Second, only a sample dish was loaded, and the indicated value was read to measure its weight. Third, the sample dish was removed, and the background was again confirmed to be consistent. Finally, the sample-loaded dish was loaded, and the sum weight of the sample and dish was read. The net weight of the sample was then derived from the difference between the read values. These measurement steps were repeated five times for each sample.

### Imaging using microcomputed tomography system and extraction of volume of interest

**Fig. 2:**
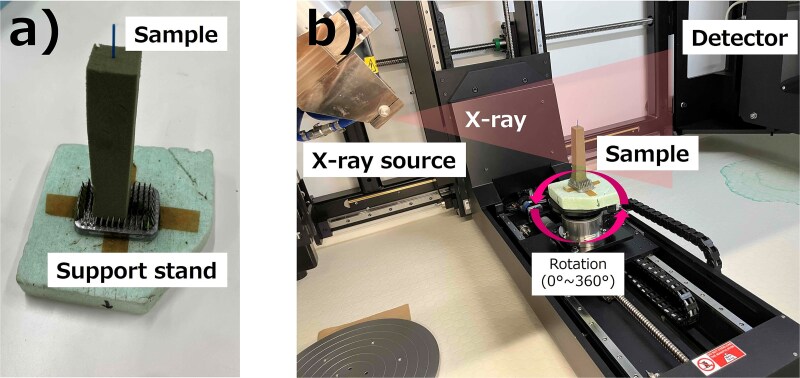
Photographs of (a) the sample fixed on a support stand and (b) experimental setup in the micro-CT imaging.

The property of the components in the detector head is summarized in Table 1. The detector head, which is comprised of a reflective coating, optical adhesive, and a small piece of Li-glass, is shaded by a graphite cap. Thus, their shape and position are invisible from the outside. The small piece of scintillator is adhered to the tip of the optical fiber using the optical adhesive, whose refractive index is quite similar to that of the scintillator to enhance the light collecting efficiency of scintillation photons. Since the scintillator is completely sunk in the drop of the optical adhesive as shown in Fig. 1, the boundary is almost invisible even though the shading cap is absent in the samples of this study. Thus, in this study, we applied a computed tomography (CT) technique with a resolution of μm (micro-CT [16]) to evaluate the micro-shape of a small piece of the scintillator. The micro-CT can distinguish the micro-shapes of the structures based on the differences of the electron density of the materials, and enables the small piece of Li-glass and the surrounding components to be distinguished. The CT images of the prepared samples were obtained by using the micro-CT system (MCT225, Nikon, Tokyo, Japan) at the Industrial Technology Center of Tochigi Prefecture. The nominal value of the maximum permissible error (MPE) in μm of the micro-CT system MCT225 was expressed as follows [17]:


(1) \begin{equation*} \mathrm{MPE}=9+\mathrm{L}/50 \end{equation*}

where L was the length of the sample in mm. Figure 2 shows the experimental setup of the micro-CT imaging. The sample was fixed using a foam material (Aquafoam, Matsumura Aqua Co., Ltd., Osaka, Japan) on the rotation stage. In MCT225, a projection image has 2000 × 2000 pixels. Each pixel size is calibrated by the manufacturer using reference samples as a function of the distance from the X-ray source to the sample stage. In the present setup, the pixel size was ⁓3 μm, which meant that the field of view had an area of ⁓6 × 6 mm^2^. The tube voltage was 105 kV and the tube current was 100 μA. The total number of projection images was 2000, and the exposure time per image was 1415 ms. A lower number of projection images might result in a less sharp image. To confirm the influence of the number of the projection images, we tested CT imaging with an increased number of projection images only for the ‘En-L’ sample. The number of projection images was increased to 3141 by an optimizing function based on the brightness on the projection image. Shading correction was conducted before every imaging.

**Table 1:** The property of the components of the detector head

Component	Material	Composition	Density [g/cm^3^]	Refractive index
Scintillator [14, 18]	Ce:Li-glass	SiO_2_ (56 wt%), MgO (4 wt%), Al_2_O_3_ (18 wt%), Ce_2_O_3_ (4 wt%), and Li_2_O (18 wt%)	2.50	1.55
Optical adhesive	NOA63		>1	1.56
Reflective coating	Titanium (IV) oxide	TiO_2_	4.23	
Shading cap	Graphite	C	2.26	

The projection images were processed using reconstruction software (VGSTUDIO, Volume Graphics GmbH, Heidelberg, Germany) to reconstruct the three-dimensional CT image. Then, the volume of interest (VOI), including the contours of the small piece of the Li-glass, was extracted based on the pixel value differences between the Li-glass and the surrounding NOA63. Finally, the extracted VOI of the small piece of the Li-glass was meshed and saved in Standard Triangulated Language (STL) format. The volume inside the contours was computed from the contoured VOI. It was then converted to mass by using a Li-glass mass density of 2.50 g/cm^3^ [14]. Through comparison of the mass computed from the VOI and the mass measured using an electronic balance, we assessed whether the shape and volume of Li-glass was appropriately reconstructed and extracted.

## RESULTS

**Fig. 3:**
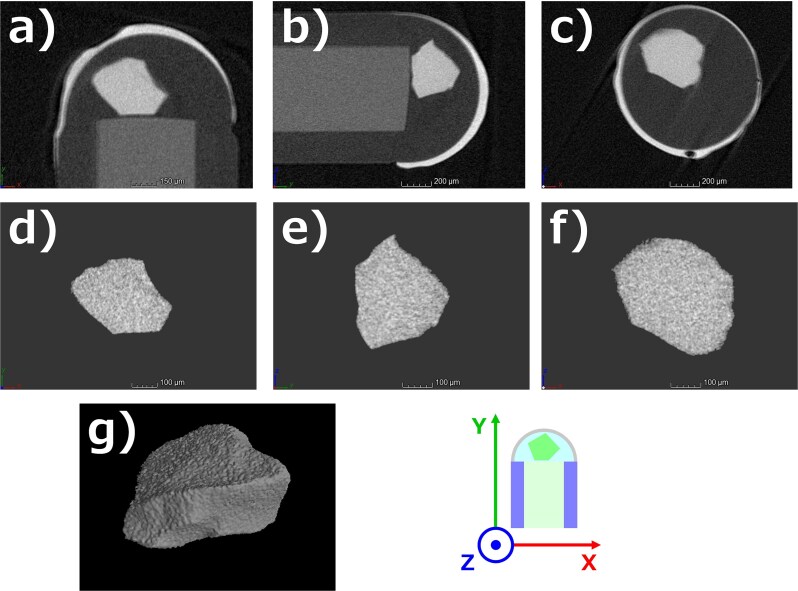
Examples of the reconstructed CT image as a 2D cross-section for the (a) x–y, (b) y–z, and (c) z–x planes, and the extracted VOI as a 2D cross-section for the (d) x-y, (e) y–z, and (f) z–x planes, and (g) 3D reconstructed volume obtained for sample ‘En-L’.

Examples of the extracted VOI from the reconstructed CT image of sample ‘En-L’ are shown in Fig. 3. The orientation of the coordinates is also shown. A random stone-like shape with defined edges on its surface was clearly visible (Fig. 3a). There were no false signals originating from the surrounding elements such as the NOA63 and the FP600URT core. Furthermore, there were no metal-artifact-like structures due to high absorption of TiO_2_, which had a mass density of 4.23 g/cm^3^ (Fig. 3b−d). These were similar for the other two samples. This result confirms that the small piece of Li-glass scintillator could be distinguished based on the pixel values in the obtained image.

The mass measured using the electronic balance XP6 and that calculated from the density and contoured VOI are listed in Table 2. The measured results were expressed as the mean and standard deviation derived from the five repeated measurements. The uncertainty of the measured mass was ˂2% for all samples (0.6%–1.8%). The mass calculated from the density and contoured VOI indicated the same trends as those measured. However, the mass from the VOI was slightly underestimated for all samples. The difference was localized within a range of 4%–6%. Increasing the number of projection images to 3141, the mass calculated from the density and contoured VOI was consistent with the mass measured by XP6 as listed in Table 3. The disagreement was diminished to 1.63%, which was equivalent to the level of the uncertainty of mass measurement by electronic balance.

**Table 2:** The mass of the small pieces of Li-glass scintillator of each sample

Sample name	Type of Li-glass	Mass measured by XP6 (mean ± standard deviation) [μg]	Mass calculated from VOI [μg]	Difference
En-L	GS20	61.4 ± 0.4	57.8	−5.86%
En-S	GS20	28.8 ± 0.4	27.7	−3.82%
Nat	GS10	31.0 ± 0.5	29.2	−5.81%

**Table 3:** The mass of the ‘En-L’ sample with the number of projection images increased to 3141

Mass measured by XP6 (mean ± standard deviation) [μg]	The number of projection images	Mass calculated from VOI [μg]	Difference
61.4 ± 0.4	2000	57.8	−5.86%
	3141	60.4	−1.63%

## DISCUSSION

The fine shape of the small piece of Li-glass scintillator was extracted from the reconstructed CT image, and the results were not affected by artifacts originating from the surrounding materials. Furthermore, the results were not affected by the differences in the size and composition of the Li-glass. However, there are some points to note in fabricating the optical fiber-based neutron detector to apply the proposed technique. First, the contours of Li-glass scintillator may be difficult to define if multiple components with high absorption are overlapped on the image. Thus, it should be noted that the small piece of Li-glass scintillator and other components with high absorption (e.g. TiO_2_ reflective coating) should be spatially separated by low-absorption materials (e.g. NOA63 optical adhesive) in the detector as shown in Fig. 3a–c. Additionally, it is possible that the air entrained in the NOA63 may form cavities around the small piece of Li-glass. Since those cavities can affect the detector response, deaeration before curing NOA63 may be important. In the present study, we found no cavity-like structures in the NOA63, thus the shape of Li-glass was expected to be the primary factor affecting the detector response.

Although the mass of the Li-glass calculated from the density and VOI was slightly underestimated, the difference was consistently within a range of 4%–6% in all samples with the number of projection images of 2000. The nominal value of MPE in μm of the micro-CT system MCT225 was expressed as Eq. (1). Assuming the grain size of Li-glass to be 300 μm, the MPE of 9.006 μm, or nearly 3%, might be considered in the present setup. It was also possible that the peripheral region of the Li-glass was partially lost in the determination process of the VOI contours. However, these regions were limited in volume, and the influence on the whole shape was considered to be small. On the other hand, the measured mass had an uncertainty of 0.6%–1.8%. It was considered that this also had little effect on the actual volume of the Li-glass. Thus, provided that the measured mass and the computed mass were in good agreement, the micro shape obtained was considered reasonable. The mass of ‘En-L’ calculated from the density and VOI reconstructed from the 3141 projection images was 60.4 μg, which was almost equivalent to the mass measured by XP6. The difference was only 1.63%, which was almost equivalent to the uncertainty of the mass measurement. This means that a sufficient number of projection images may mitigate the differences between measured and calculated masses. Consequently, through the comparison of the mass measured using the electronic balance and that computed from the micro-CT image, the micro shape of Li-glass was concluded to be properly reconstructed.

In the neutron field of an accelerator-based neutron source for boron neutron capture therapy, typical neutron flux at the beam exit port reaches nearly 10^9^ n/cm^2^/s, and thermal neutron flux reaches ˃10^9^ n/cm^2^/s in a water phantom for quality assurance and quality control of the neutron source [19–21]. Since the thermal neutron sensitivity of the optical fiber-based neutron detector using ^6^Li-enriched Ce:Li-glass is typically in the order of 10^−4^ cm^2^ [8, 22], the detector counting rate is expected to be larger than 10^5^ counts per sec. Assuming that the statistical uncertainty of the detector count is 0.3%, to determine the neutron fluence with an uncertainty of ˂5%, the systematic uncertainty of the detector must be ˂4.99%. As mentioned above, the uncertainty of the micro shape of the scintillator was suppressed to 1.63% by increasing the number of projection images. On the other hand, the variation of the detection efficiency resulted from the uncertainty in the scintillator shape is estimated to be ˂30% from the results of the calculation for the virtual shapes (Supplementary Document Table S1 and Fig. S1). However, the detection efficiency can be corrected with considerable accuracy by determining the accurate scintillator shape. For example, incorporating the actual scintillator shape obtained as an STL file, which can be easily imported into the simulation, into Monte Carlo simulation, the detection efficiency corresponding to the direction of incident neutrons can be calculated, and the detector response can be corrected. Thus, it will be possible to diminish the systematic uncertainty of the detector ˂4.99% provided that the detector response is properly corrected based on the micro shape of the Li-glass finely obtained by our proposed technique.

In conclusion, we applied the micro-CT technique to obtain the fine structure of a small piece of Li-glass used in the optical fiber-based neutron detector. In the obtained results, the random shape with defined edges was clearly observed. The small piece of Li-glass was completely separated from the surrounding materials based on pixel values. The mass calculated from the density and volume of the Li-glass contoured on the CT image was in good agreement with that measured by the electronic balance with a readability of 1 μg. According to the ICRU24 report, the overall dose uncertainty delivered to a patient during X-ray radiotherapy must be ˂5% [13]. The proposed technique can deduce the micro shape of Li-glass with an accuracy equivalent to that of the mass measurement. The proposed technique will be useful in the evaluation and correction of the detector response, in particular when high accuracy is needed, such as for medical applications (e.g. quality control of the neutron sources for BNCT [23–25]). In addition, it will be applicable to detectors using other types of ^6^Li-based neutron scintillators, such as Eu:LiCaAlF_6_ crystal [10] and LiF/Eu:CaF_2_ eutectic [26]. In the future, we will investigate the factors that affect the light collection efficiency of the detector (e.g. the shape or amount of the optical adhesive). Furthermore, we will perform experiments and simulation to evaluate the shape-dependent effects on the detector response, such as a directional dependence and self-shielding effect. We will import the STL data obtained of the Li-glass’s micro shape into Monte Carlo simulation code and perform a detector-specific calculation. Through analysis of these results, it will be possible to propose a method to correct the detector response.

## Supplementary Material

010_Supplemenatry_Document_ver250530_v2_rraf048
