# Supplementary material for: Three-dimensional reconstruction of a small piece of Ce-doped lithium glass scintillator of an optical fiber-based neutron detector using microcomputed tomography technique
Source: J Radiat Res. 2025 Jul 29;66(5):563–9. doi: 10.1093/jrr/rraf048 (PMC12460048; doi:10.1093/jrr/rraf048)
Supplement: 010_Supplemenatry_Document_ver250530_v2_rraf048 [file 010_supplemenatry_document_ver250530_v2_rraf048.docx]

Supplementary Document:

To estimate the variation in detection efficiency attributed from the differences in the scintillator shapes, the directional dependence of the reaction rate in some representative shapes of small piece of Li-glass scintillator was computed using Monte Carlo simulation by PHITS. PHITS Ver. 3.33 was used.

Since the small pieces of Li-glass scintillator used in this study have grain size of 212−400 μm, a sphere with a diameter of 300 μm was assumed as a reference of the detection efficiency. Then, we assumed the two representative shapes with volumes equal to the sphere as a cube and a cuboid. The cube had a side length of 242 μm and the cuboid had a height of 600 μm, which was equal to the core diameter of FP600URT, and a bottom surface with an area of 153 × 153 μm^2^. The cuboid is a possible shape which is expected to express the most extreme variation in detection efficiency. The representative shapes of Li-glass scintillator assumed were summarized in Table S1. The directional dependence of ^6^Li(n,t)α reaction rates in these Li-glass scintillator was computed for neutrons with energy of 25 meV for a range of 0°−330° by 30°.

Figure S1 shows the directional dependence of detection efficiency of the representative shapes. The results were shown as a relative value to the efficiency of the sphere. From the results, as expected, the cuboid had the maximum variation in detection efficiency, and its variation range was from −24% to +5% (Table S1). Thus, the variation in detection efficiency was estimated to have a magnitude of approximately 30% or less.

Table S1. Representative shapes of Li-glass scintillator assumed to estimate the variation in detection efficiency

| Shape | Size specification | Range of variation in efficiency |
| --- | --- | --- |
| Cube | 242 × 242 × 242 μm^3^ | −2% to +0% |
| Cuboid | 153 × 153 × 600 μm^3^ | −24% to +5% |


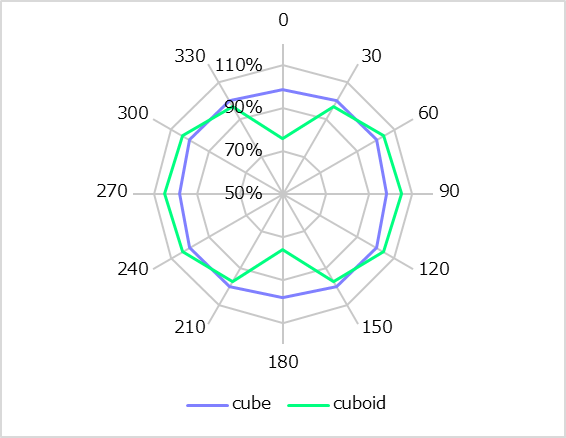


Fig. S1. Results of the calculation of the directional dependence of the detection efficiency for each representative shape of Li-glass scintillator
